# Pathomechanisms and therapeutic opportunities in radiation-induced heart disease: from bench to bedside

**DOI:** 10.1007/s00392-021-01809-y

**Published:** 2021-02-16

**Authors:** Márta Sárközy, Zoltán Varga, Renáta Gáspár, Gergő Szűcs, Mónika G. Kovács, Zsuzsanna Z. A. Kovács, László Dux, Zsuzsanna Kahán, Tamás Csont

**Affiliations:** 1 MEDICS Research Group, Department of Biochemistry, Interdisciplinary Center of Excellence, University of Szeged, Szeged, 6720 Hungary; 2 Department of Oncotherapy, Faculty of Medicine, University of Szeged, Szeged, 6720 Hungary; 3 Department of Biochemistry, Faculty of Medicine, University of Szeged, Szeged, H-6720 Hungary

**Keywords:** Onco-cardiology, Radiation heart sequelae, Molecular pathomechanisms of radiation-induced heart disease, Prevention and therapy of radiation-induced heart disease

## Abstract

Cancer management has undergone significant improvements, which led to increased long-term survival rates among cancer patients. Radiotherapy (RT) has an important role in the treatment of thoracic tumors, including breast, lung, and esophageal cancer, or Hodgkin's lymphoma. RT aims to kill tumor cells; however, it may have deleterious side effects on the surrounding normal tissues. The syndrome of unwanted cardiovascular adverse effects of thoracic RT is termed radiation-induced heart disease (RIHD), and the risk of developing RIHD is a critical concern in current oncology practice. Premature ischemic heart disease, cardiomyopathy, heart failure, valve abnormalities, and electrical conduct defects are common forms of RIHD. The underlying mechanisms of RIHD are still not entirely clear, and specific therapeutic interventions are missing. In this review, we focus on the molecular pathomechanisms of acute and chronic RIHD and propose preventive measures and possible pharmacological strategies to minimize the burden of RIHD.

## Introduction

Cardiovascular and cancerous diseases are the leading causes of mortality worldwide [1, 2]. The most common cancerous diseases are thoracic malignancies, including lung and breast cancers among adults [2]. Recently, cancer management has undergone significant improvement, leading to increased long-term survival rates among cancer survivors. Consequently, age-related chronic diseases and cardiovascular risk factors, including hypertension, diabetes mellitus, hyperlipidemia, chronic kidney disease, and smoking, are often aggravated by chronic side effects of multimodality cancer therapy, accelerating the progression of atherosclerosis and increasing the burden of cardiovascular diseases (CVDs) in cancer survivors [3].

While high-energy ionizing radiation successfully destroys cancer cells, at the same time, it may have harmful effects on the surrounding healthy tissues leading to various side effects [4]. RT has been frequently used in thoracic malignancies, including breast, lung, esophageal cancer, thymoma, and Hodgkin's lymphoma, which could be in close anatomical proximity to the heart [5]. Depending on the RT technique and dose, the heart may be at risk of being exposed to ionizing radiation resulting in radiogenic sequelae in a dose-dependent manner [6]. The syndrome of unwanted cardiovascular side effects of thoracic RT is called radiation-induced heart disease (RIHD), and the risk of development of RIHD is a critical concern in current oncology practice. RIHD includes structural and functional abnormalities of the pericardium, coronary vessels, myocardium, valves, and conduction system [7, 8]. Much of our current knowledge on radiation-induced cardiovascular complications in cancer survivors is based on the patients' data coming from the era of the 1980s or before that, with less developed RT techniques, extended RT fields, and high radiation doses [4, 9]. The earliest data indicating the presence of RIHD originate from follow-up studies of lymphoma and breast cancer patients due to the high incidence and high cure rate of these diseases [10, 11]. In clinical practice, the consequences of RIHD mostly emerge in breast cancer patients receiving left-sided postoperative RT and less frequently in esophageal cancer patients treated with preoperative chemo-radiotherapy [6].

The prevention or management of radiogenic CVDs has become a challenge in clinical practice since RIHD can worsen the outcome, quality of life, and health care costs in long-term cancer survivors [12, 13]. These factors have recently contributed to the emergence of a new specialty termed onco-cardiology or cardio-oncology. Unfortunately, therapeutic options for RIHD are currently insufficient. Therefore, understanding the exact molecular mechanisms in the progression of RIHD is necessary for developing preventive and therapeutic strategies without attenuating the effect of RT on cancer cells. Tailored surveillance of patients according to their risk status serves early intervention if necessary [12, 13].

In this review, we summarize our current knowledge on the molecular pathomechanisms of the development and progression of RIHD and overview those potential pharmacological and other strategies that may be suitable for the prevention or management of RIHD.

## Clinical manifestations of RIHD

**Fig. 1:**
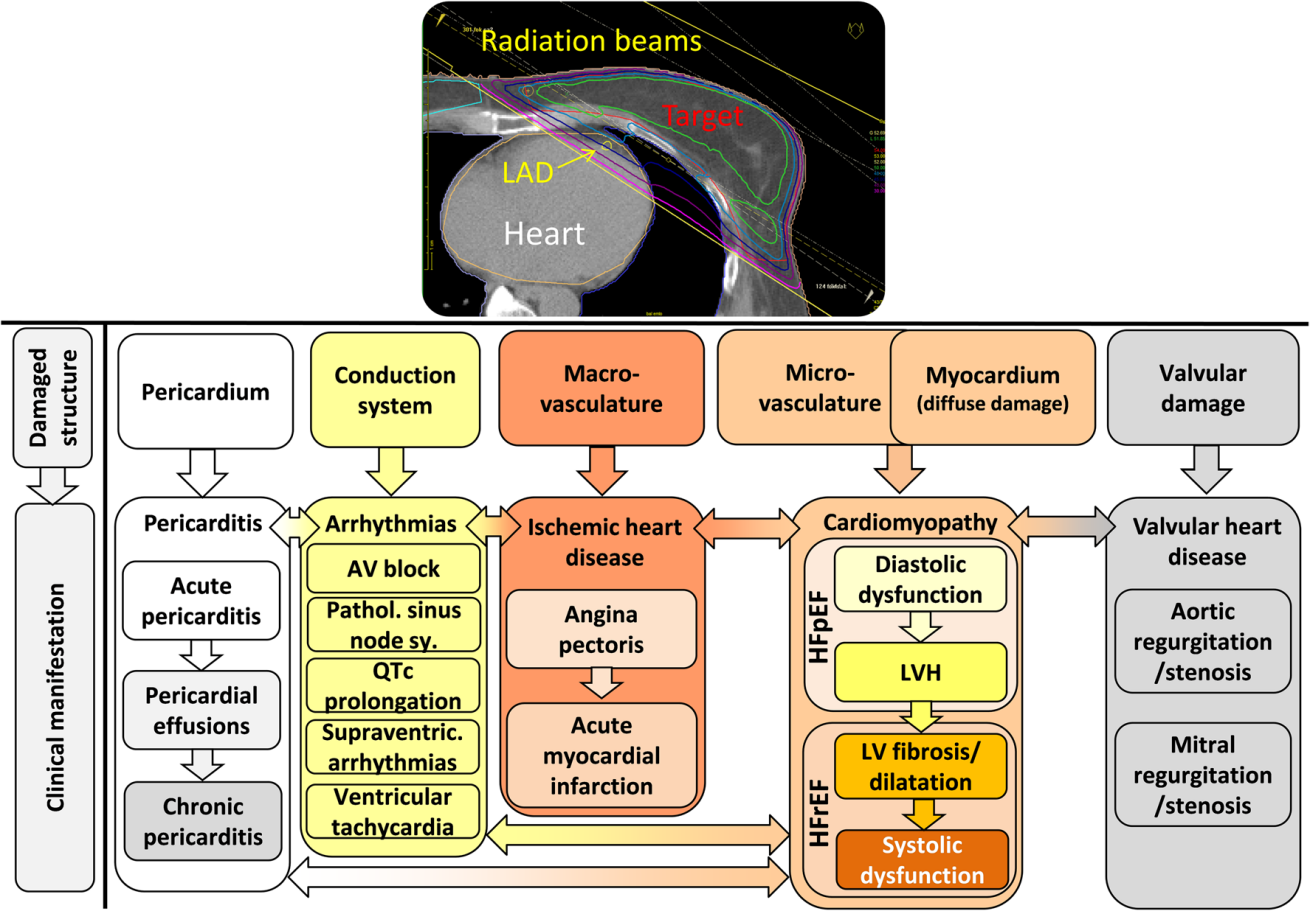
Possible clinical manifestations of RIHD. RIHD is a progressive disease that covers a broad spectrum of cardiac pathology. RIHD may manifest as acute or chronic pericarditis, conduction system abnormalities, ischemic heart disease, cardiomyopathy, heart failure including HFpEF and HFrEF, or valvular heart disease, according to the site of damage. *LAD* left descending coronary artery, *LVH* left ventricular hypertrophy, *HFpEF* heart failure with preserved ejection fraction, *HFrEF* heart failure with reduced ejection fraction

RIHD may manifest in a broad spectrum of cardiovascular complications, including acute and chronic pericarditis, ischemic heart disease (IHD), cardiomyopathy and heart failure (HF), valvular heart disease, and cardiac conduction abnormalities [4, 14] (Fig. 1). Complications typically appear years to decades after the irradiation, showing a median of 10–15 years. The overall absolute risk of cardiac death is related to the mean heart dose (MHD) of RT [9, 15]. Radiation-induced cardiovascular complications are more severe with (i) higher total radiation dose [6], (ii) extended target volume exposure with closer tumor localization to the heart [16], (iii) younger age at the radiation exposure [9, 15], (iv) longer follow-up duration [17], (v) concomitant therapy with cardiotoxic chemotherapeutic drugs such as anthracyclines and biological agents [18, 19], (vi) presence of genetic factors, and (vii) comorbidities and cardiovascular risk factors [3].

### Acute forms of RIHD

#### Acute pericarditis

Acute pericarditis could be the earliest form of RIHD [20] (Fig. 1). Before the era of selective RT-techniques in the late 1980s, 80% of patients receiving thoracic irradiation suffered from acute pericarditis [20]. In these cases, high MHD (> 36 Gy) administered to > 30% of the heart was responsible for the development of acute exudative pericarditis [5, 20]. Nowadays, its occurrence is rare (< 5%) due to alertness and the modern heart-sparing RT-techniques [13, 21]. Acute pericarditis causes chest pain, fever, and ECG abnormalities. Approximately half of the affected patients suffer from hemodynamic abnormalities due to significant pericardial effusion. Even cardiac tamponade can develop necessitating intervention [20].

#### Acute conduction system abnormalities

In the acute phase, some patients develop mostly reversible asymptomatic ECG repolarization abnormalities, which rarely persists (the incidence is unknown due to the lack of screening during or shortly after RT) [14]. These events may occur in response to transitory circulatory/metabolic changes, or if irradiation induces the development of definitive structural changes in the conduction system (e.g., via increasing oxidative/nitrosative/nitrative stress or inflammation), that probably affects an irrelevant part of the heart only.

### Chronic forms of RIHD

#### Chronic pericarditis

The presence of pericardial effusion in the acute phase might predispose patients to chronic pericarditis of delayed onset from months to years [14] (Fig. 1). Certain chemotherapeutic drugs (e.g., anthracyclines, cyclophosphamide, and bleomycin) may enhance the risk of radiogenic pericarditis development [20]. Up to 20% of patients develop chronic constrictive pericarditis with severe symptoms requiring pericardiectomy at a median time of 11 years post-irradiation [22].

#### Ischemic heart disease (IHD)

Our understanding concerning the burden of radiogenic IHD is based the most on epidemiological studies [6, 8, 9, 11, 15]. Nevertheless, estimations may be given using normal tissue complication probability models [23]. However, prospectively collected data with careful follow-up observations are needed [24]. In the various studies, different end-points, dose parameters, and statistical methods were used (Table 1). The studied populations also differed: some included all irradiated patients [8, 9, 11, 15] while others, only patients with major IHD events such as the need for coronary intervention/acute myocardial infarction (AMI) or IHD-related death [25, 26]. The risk of coronary artery disease (CAD) is clearly radiation dose-dependent. The traditional reference dose parameter is MHD [8, 9]. Nevertheless, Bogaard et al. showed that among other dose parameters, the volume of the left ventricle receiving at least 5 Gy (LV-V5) is the most sensitive predictor of coronary events [27]. Most studies agree that beyond radiation dose, risk factors for CAD or the history of IHD, and young age are also determinants of outcome (Table 1) [8, 9, 11, 15, 27].

**Table 1:** The effect of radiation dose on the risk of coronary artery disease (CAD): studies with various dosimetry data

Type of study	Radiotherapy era and number of cases	Dosimetry data	Related risk	Dosimetry data	Related risk	Effect of age	Effect of CAD risk factors	Refs.
Population-based case–control study endpoint: major coronary event*	Stockholm 1958–2002 Denmark 1977–2007 *n* = 963 cases	MHD = 1 Gy increase	ERR = 7.4%	MHD = 2 Gy MHD = 2–4 Gy MHD = 5–9 Gy MHD ≥ 10 Gy	10% (95% CI, − 9–33), 30% (95% CI, 14–49), 40% (95% CI, 15–72),	Not reported	ERR(IHD) = 6.67 (ERR_<10yrs_ = 13.43 ERR_>10 yrs_ = 2.09) ERR(other) = 1.96 (ERR_<10yrs_ = 2.60 ERR_>10 yrs_ = 1.63)	[8]
Cohort study endpoint:acute coronary event	2005–2008 *n* = 910 breast cancer patients	MHD = 1 Gy increase	Cumulative incidence = 16.5%	LV-V5 = 1 Gy increase	HR = 1.017	Increased with age	Increased with their presence	[27]
Nested case–control study, endpoint: AMI	1970–2009 *n* = 183 cases (median MHD: 8.9 Gy)	MHD = 1 Gy increase	ERR = 6.4%	MHD > 20 Gy	3.4 × increased risk	ERR_<45 yrs_ = 24.2%/Gy, ERR_≥50 yrs_ = 2.5%/Gy	ERR1 _1 risk factor_ = 1.86	[15]
Retrospective chort study endpoint: OS	1998–2012 Mayo Clinic, *n* = 76 pts with breast, lung, mediastinum, GI tumors, RT and cardiac stenting	MHD (Gy)	HR (Cox multivariate analysis) = 1.05 (95% CI 1.02–1.08)	ACEI/ARB use	HR (Cox multivariate analysis) = 3.04 (95% CI 1.28–7.19)	None in multivariate analysis	None in multivariate analysis	[26]
Nested case–control endpoint: CAD	1965–1995 *n* = 325 HML cases	MHD = 1 Gy increase	ERR = 7.4%	MHD = 20 Gy	2.5 × increased risk	ERR_<27.5yrs_ = 20.0% ERR_27.5–36.4yrs_ = 8.8% ERR_36.5–50.9 yrs_ = 4.2%;	ERR_1 risk factor_ = 1.5 ERR_physical activity_ = 0.5	[9]

*ACEI* ACE inhibitor, *AMI* acute myocardial infarct, *ARB* angiotensin receptor blocker, *CAD* coronary artery disease, *ERR* excess rate ratio, *HR* hazard ratio, *IHD* ischemic heart disease, *HML* Hodgkin lymphoma, *LV-V5* the volume of the left ventricle receiving at least 5 Gy, *MHD* mean heart dose, *OS* overall survival, *RR* relative risk, *Major coronary event: AMI/coronary revascularization/death from IHD

Breast cancer patients receiving radiation doses of < 2 Gy, 2–4 Gy, 5–9 Gy, and > 10 Gy had a dose-dependent excess risk for angina pectoris, AMI, and sudden cardiac death of 10%, 30%, 40%, and 116%, respectively [8, 9, 27] (Table 1; Fig. 1). The risk for IHD increased linearly with the MHD by 7.4% per 1 Gy absorbed dose with no apparent lower threshold [8]. Similar dose–response results were found by Jacobse et al. in a nested case–control study: every 1 Gy increase in MHD was associated with a 6.4% increase in the risk of AMI; in MHD > 20 Gy cases, the risk of AMI was 3.4 times higher (Table 1) [15]. Based on modern three-dimensional conformal radiation therapy (3DCRT) dose-volume data, Bogaard et al. also confirmed these results. They introduced the LV-V5 parameter indicating a 1.7% increase in coronary events incidence by every 5 Gy increase of dose to the left ventricle [27]. Among lymphoma patients, identical results were found on MHD data and long-term AMI incidence as in the classic Darby-study; in MHD > 20 Gy cases, 2.5 times higher AMI risk was detected [9]. The dose-dependent and irradiated volume-dependent nature of RIHD-related survival were demonstrated in a retrospective cohort study in lymphoma patients with CAD requiring coronary intervention [26]. The strongest predictor of a major coronary event is left-sided RT [28, 29]. Boekel et al. showed a significantly increased risk of IHD when the chest wall or the internal mammary nodes were irradiated independent of laterality in breast cancer patients [16]. The association between the radiation dose to a cardiac segment and the injury of the affected structure was demonstrated by Taylor et al. [30]. In this study, the highest RT doses were detected in the distal part of the left anterior descending (LAD) artery [30].

#### Cardiomyopathy and heart failure (HF)

Radiation-induced cardiomyopathy and consequent HF are progressive multifactorial diseases, which may evolve over several years (Fig. 1). They are often aggravated by medical anti-cancer therapies, concomitant radiation-induced valvular heart disease (VHD), or IHD. RT-induced cardiomyopathy and HF cover a spectrum of cardiac pathological conditions among which a typical initial phase is HF with preserved ejection fraction (HFpEF). HFpEF is characterized by diastolic dysfunction and compensatory left ventricular hypertrophy (LVH) (Fig. 1). [31–34]. Later on, progressive interstitial fibrosis develops, separating and replacing the cardiomyocytes, which ultimately results in HF with reduced ejection fraction (HFrEF) [20, 35]. A tolerance dose of 40 Gy has been estimated for the human myocardium for the end-point of diffuse myocardial injury [20]. Indeed, diffuse myocardial injury is more common in patients who have received higher RT doses (> 60 Gy) and/or anthracycline chemotherapy [14, 29]. Radiation-induced cardiomyopathy is often asymptomatic. Its risk increases 5 years after RT but may develop even decades after RT [20].

#### Valvular heart disease (VHD)

Within the first 10 years after RT, the earliest morphological changes appear to be leaflet thickening, fibrosis, shortening and calcification, and consequent regurgitation preferentially at the mitral or aortic valves [20] (Fig. 1). The progression to fibrotic thickening and calcification of the valves may lead to stenosis, which develops mainly in the aortic valve approximately 20 years after RT [20]. The rate of VHD correlates better with the RT dose to the affected [36] valve than to the mediastinal dose [37]. Myocardial ischemia and hypoxia caused by IHD and fibrosis also play a role in the development of VHD that may contribute to HF [14].

#### Chronic conduction system abnormalities

In the chronic phase after irradiation, in about 5% of the cases, radiogenic conduction system abnormalities develop [37, 38]. Among these, bundle branch blocks and first-degree atrioventricular (AV) block occur most commonly [39], but pathological sinus node syndrome, QTc prolongation, supraventricular arrhythmias, and ventricular extrasystole or tachycardia may also develop [14, 39, 40] (Fig. 1). Circulatory changes such as autonomic dysfunction with tachycardia and blunted blood pressure, rarely syncope or even sudden death related to the denervation-like status of the heart may occur; (nevertheless, similar symptoms due to neck irradiation with injury of the vessels and baroreceptors should be distinguished) [38, 41, 42].

Late conduction anomalies may be explained with various pathomechanisms. Most obviously, fibrotic lesions resulted from decreased microvessel density, chronic hypoxia, compensatory hypertrophy are behind the abnormalities [35, 43–46]. In other cases, RT-related valvular disease and increased right atrial pressure cause atrial arrhythmias [38], or exercise-induced ischemia of the atrioventricular node due to the stenosis of the right coronary artery results in AV block [40, 47].

## Pathomechanisms of RIHD

**Fig. 2:**
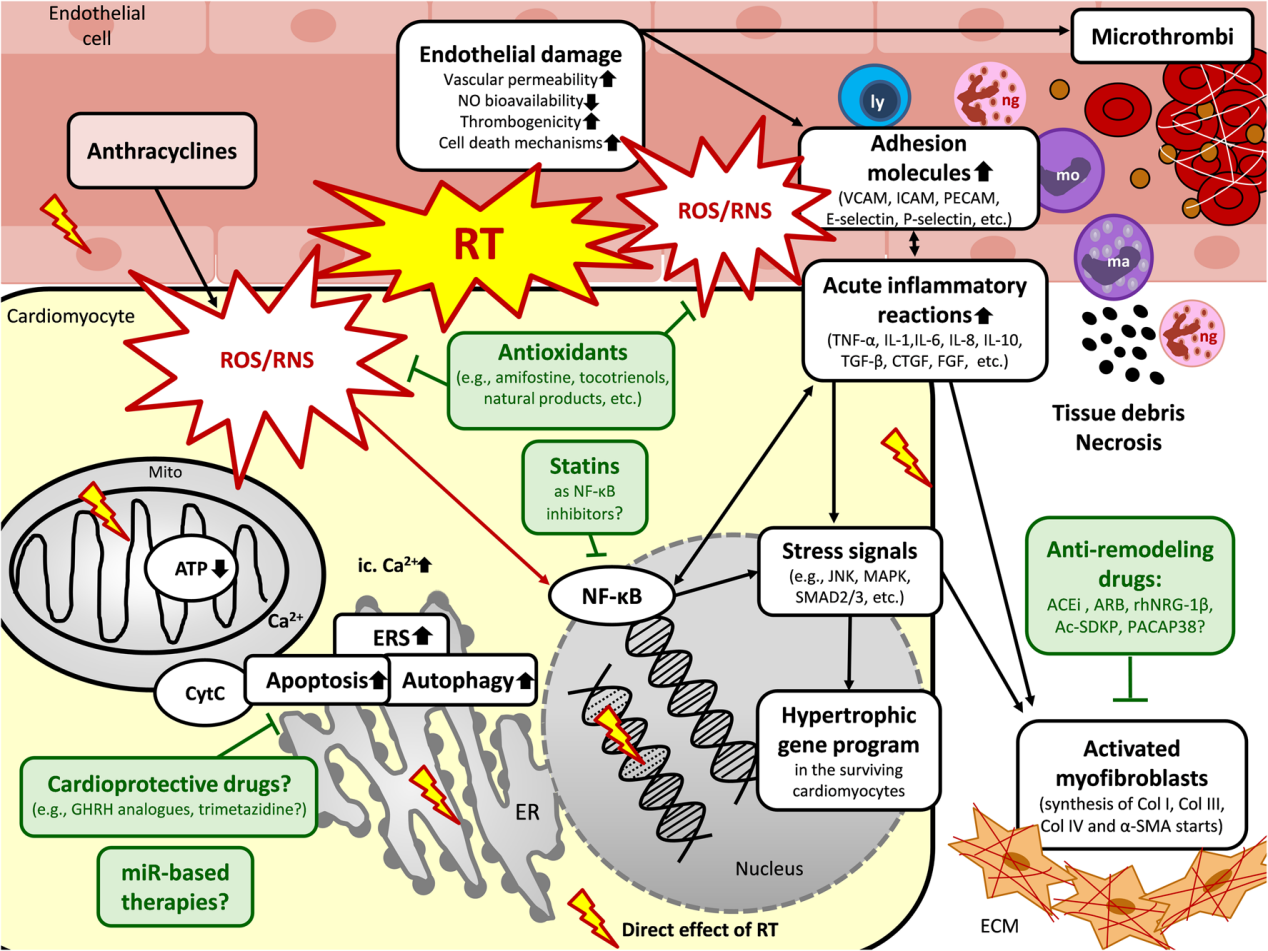
Putative mechanisms in the acute phase of RIHD and potential pharmacological interventions. RT could induce immediate oxidative/nitrosative/nitrative damage of macromolecules, including DNA, proteins, and lipids, in all cardiac cell types. The increased oxidative/nitrative/nitrosative stress triggers other biological processes, including acute inflammation, and cell death forms in the acute phase of RIHD in the different cell types. Parallel, hypertrophic, and fibrotic gene programs start in the surviving cardiomyocytes as compensatory mechanisms. Potential preventive and therapeutic pharmacologic agents are depicted in green boxes targeting different molecular mechanisms. *ACEi* angiotensin-converting enzyme inhibitors, *Ac-SDKP* N-acetyl-Ser-Asp-Lys-Pro, *ARB* angiotensin receptor blockers, *α-SMA* α-smooth muscle actin, *ATP* adenosine triphosphate, *Ca*^*2+*^ calcium ion, *Col* collagen, *CTGF* connective tissue growth factor, *CytC* cytochrome C, *ERS* endoplasmic reticulum stress, *FGF* fibroblast growth factor, *GHRH* growth hormone-releasing hormone, *ICAM* intercellular adhesion molecules, *IL* interleukin, *JNK* c-Jun N-terminal kinases, *ly* lymphocyte, *ma* macrophage, *MAPK* mitogen-activated protein kinase, *miR* microRNA, *mo* monocyte, *Mito* mitochondrion, *NF-κB* nuclear factor-κB, *ng* neutrophil granulocyte, *NO* nitric oxide, *PACAP38* pituitary adenylate cyclase-activating polypeptide 38, *PARP1*poly-ADP-ribose-polymerase 1, *PECAM* platelet endothelial cell adhesion molecule, *rhNRG-1β* recombinant human neuregulin-1β, *ROS/RNS* reactive oxygen and nitrogen species, *RT* radiotherapy, *TGF-β* tissue growth factor-β, *TNF-α* tumor necrosis factor- α, *VCAM* vascular cell adhesion molecule

**Fig. 3:**
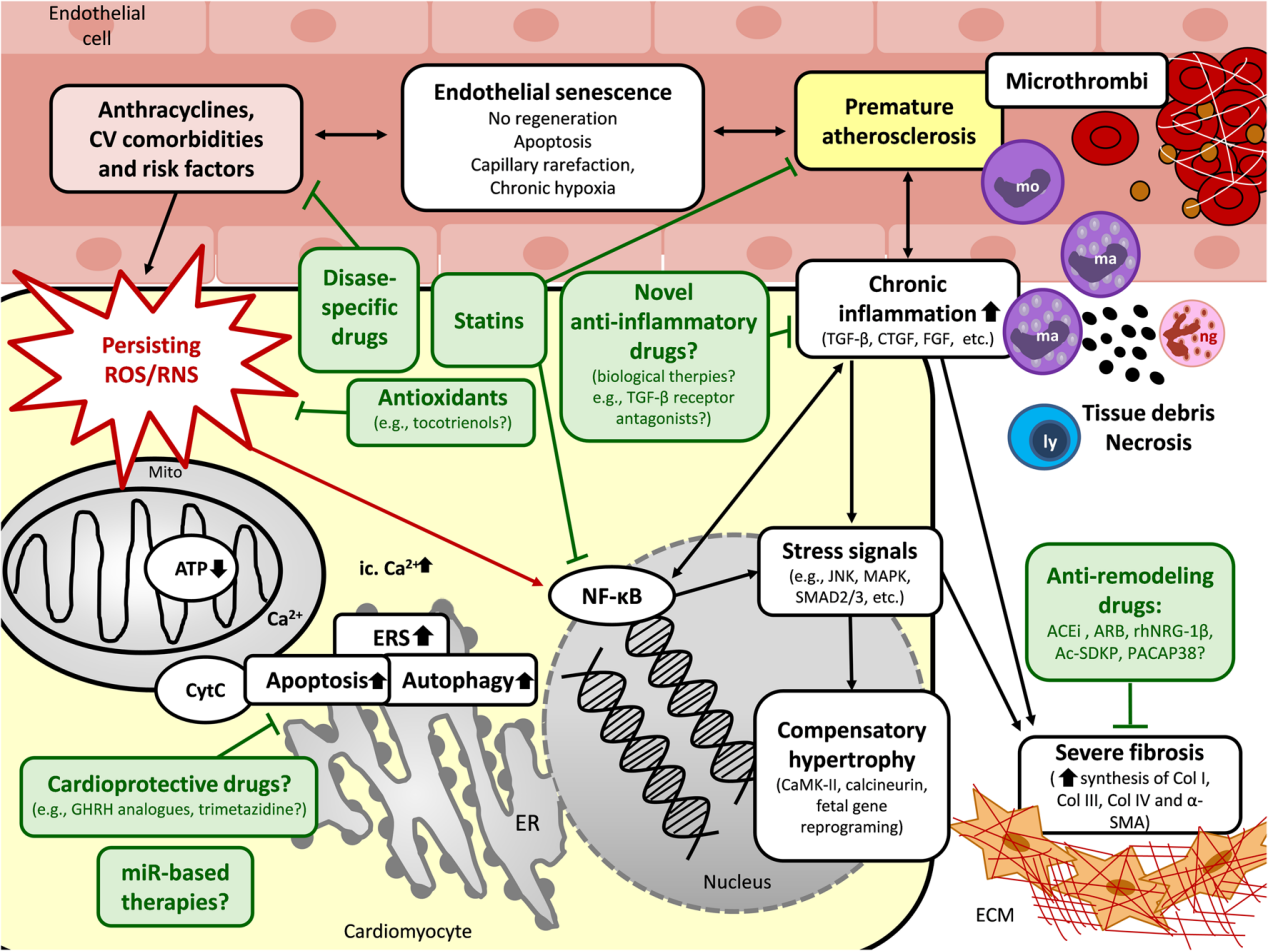
Putative mechanisms in the chronic phase of RIHD and potential pharmacological interventions. Several pathomechanisms in the chronic phase of RIHD including oxidative/nitrative/nitrosative stress, cell death, and inflammatory processes, overlap during the acute and chronic phases of RIHD. These mechanisms could activate and potentiate each other in the different cardiac cell types leading to a vicious cycle. In the early chronic phase of RIHD, compensatory mechanisms including manifest left ventricular hypertrophy and endothelial cell proliferation are predominant. If these compensatory mechanisms are exhausted, fibrosis and endothelial senescence play the central role in the late phase of disease progression. The exact molecular transition points from acute to compensated and decompensated chronic forms of RIHD are unknown yet. Potential preventive and therapeutic pharmacologic agents are depicted in green boxes targeting different molecular mechanisms. ACEi angiotensin-converting enzyme inhibitors, *ARB* angiotensin receptor blockers, *α-SMA* α-smooth muscle actin, *ATP* adenosine triphosphate, *Ca*^*2*+^ calcium ion, *CaMK* Ca^2+^/calmodulin-dependent protein kinase, *CKD* chronic kidney disease, *Col* collagen, *CTGF* connective tissue growth factor, *CytC* cytochrome C, *CV* cardiovascular, *ERS* endoplasmic reticulum stress, *ETC* electron transport chain, *FGF* fibroblast growth factor, *GHRH* growth hormone-releasing hormone, *JNK* c-Jun N-terminal kinases, *ly* lymphocyte, *ma* macrophage, *MAPK* mitogen-activated protein kinase, *miR* microRNA, *mo* monocyte, *Mito* mitochondrion, *NF-κB* nuclear factor-κB, *ng* neutrophil granulocyte, *PACAP38* pituitary adenylate cyclase-activating polypeptide 38, *PARP1* poly-ADP-ribose-polymerase 1, *PECAM* platelet endothelial cell adhesion molecule, *ROS/RNS* reactive oxygen and nitrogen species, *TGF-β* tissue growth factor-β, *TNF-α* tumor necrosis factor-α

RIHD is a progressive multifactorial disease that has overlapping common and different molecular pathways in the acute and chronic phases. RT simultaneously causes damage to the macrovasculature (i.e., coronary arteries), the microvasculature, and the myocardium (i.e., diffuse injury) [7]. Key questions relating to the precise molecular mechanisms of the disease progression in RIHD from acute to chronic heart diseases remained unanswered. The chain of biological events from acute to chronic forms is more likely a complex interaction between molecular processes. A substantial body of evidence suggests that the radiation-induced immediate oxidative/nitrosative/nitrative damage of macromolecules, including DNA, proteins, and lipids, is the initiating event in RIHD. At this early phase, the increased oxidative/nitrative/nitrosative stress triggers other biological processes, including endothelial cell injury, acute inflammation, and the various forms of cell death [35, 43, 44] (Fig. 2). In the early chronic phase of RIHD, several compensatory mechanisms, including endothelial cell proliferation and cardiac hypertrophy, develop in the sublethally damaged surviving cells [35, 43, 44] (Fig. 3). If these compensatory mechanisms are exhausted, chronic inflammatory processes, fibrosis, and endothelial senescence play the central role in the disease progression [35, 43, 44] (Fig. 3). The exact molecular transition mechanisms and time points between the acute to compensated or decompensated chronic forms of RIHD are yet unknown. Moreover, several pathomechanisms, including oxidative/nitrative/nitrosative stress, cell death, and inflammatory processes, overlap during the acute and chronic phases of RIHD. These mechanisms could activate and potentiate each other leading to a vicious cycle in the RIHD progression. Therefore, the precise understanding of the complex interplay of these acute and chronic molecular mechanisms would help to develop strategies to counteract the progression of RIHD. In this section, we briefly summarize the predominating pathomechanisms in which have a role in developing the acute and chronic forms of RIHD.

### Pathomechanisms in the acute phase of RIHD

#### Mechanisms of increased oxidative/nitrosative/nitrative stress in the acute phase of RIHD

Increased oxidative/nitrosative/nitrative stress plays a crucial role in developing both the desired anticancerous effects and the undesired side effects of RT. Absorption of ionizing radiation used for RT may induce both direct and indirect effects in all cell types [48] (Fig. 2). Ionizing radiation can directly disrupt atomic structures, leading to further chemical and biological changes [49] (Fig. 2). Approximately 80% of the cells is water. Therefore, the initial radiation-induced cellular damage is mostly caused by the direct radiolysis of water generating reactive species leading to indirect effects [48]. The major reactive species produced in the radiolysis of water are superoxide (O_2_^•−^), hydroxyl radical (^•^OH), electrons (e^−^), and hydrogen peroxide (H_2_O_2_). Organic radicals (R^•^) are also formed by H-abstraction reactions initiated by ^•^OH radicals. These carbon-centered radicals usually react rapidly with O_2_ to give peroxyl radicals (RO_2_^•^), which are stronger oxidizing agents than their parent radicals. The RO_2_^•^ radicals can abstract H^•^ from other molecules to form hydroperoxides (ROOH) [48]. Ionizing radiation-induced tissue injury may up-regulate inducible nitric oxide synthase (iNOS), thereby generating a large amount of nitric oxide (^•^NO), which can react with O_2_^•−^ to form peroxynitrite (ONOO^−^) and secondarily other reactive nitrogen species (RNS) [48–50] (Fig. 2). Other enzymatic sources for reactive oxygen species (ROS) include NADPH oxidases (NOX isoforms), lipoxygenases (LOX), cyclooxygenases (COX), peroxidases in inflammatory cells, and xanthine oxidase, which can be activated by RT-induced tissue injury [35, 48]. Ionizing radiation may also disrupt the mitochondrial respiratory chain contributing to persistent oxidative/nitrosative/nitrative stress. The removal of ROS/RNS during RT via the key antioxidant enzymatic systems, including, e.g., superoxide dismutase (SOD), catalase, glutathione peroxidase, glutathione reductase, and heme oxygenase, may also be insufficient [48]. Accumulated ROS/RNS during and shortly after irradiation may cause macromolecular damage including, lipid peroxidation, protein oxidation/nitration, inactivation of enzymes, DNA damage, interaction with both DNA repair enzymes (e.g., poly-ADP-ribose polymerase 1 [PARP1], p53) and transcription factors (e.g., nuclear factor-κB [NF-κB]) [14, 20, 35, 43, 48, 51] (Fig. 2). Oxidative/nitrative stress can also induce acute inflammation and cell death via different mechanisms. The macromolecular and cellular damage may result in the activation of the inflammatory response (interleukins [IL] including, e.g., IL-1, IL-6, IL-8, IL-10, tumor necrosis factor-alpha [TNF-α], and transforming growth factor-beta [TGF-β]), stress signals (e.g., c-Jun N-terminal kinase [JNK], and p38-MAPK) or cell death (e.g., apoptosis and necrosis), and dysregulation of autophagy. The oxidation/nitration of proteins involved in excitation–contraction coupling, contractility, Ca^2+^-handling, elements of the mitochondrial electron transport chain and Krebs cycle, metabolism, and extracellular matrix might result in acute and chronic deleterious events [14, 20, 35, 43, 48, 51] (Fig. 2).

#### Endothelial cell injury and acute inflammation in RIHD

Endothelial cell injury is considered the primary cause of radiation damage in cardiac tissue [14, 35] (Fig. 2). Within minutes after RT, increased vascular permeability and vasodilation are present [5, 35, 43]. In the first few hours and days after RT, activated NF-κB [52, 53] may induce the secretion of adhesion molecules including, e.g., E-selectin [54], P-selectin [54], intercellular adhesion molecule-1 (ICAM-1) [54], vascular cell adhesion molecule-1 (VCAM-1) [55], platelet endothelial cell adhesion molecule-1 (PECAM-1) [55], and cytokines (e.g., IL-6, IL-8) [56, 57] in the damaged endothelial cells thereby activating leukocyte rolling, arrest, and transmigration [35, 43, 52]. The predominant inflammatory cells in the acute phase are neutrophil granulocytes. They infiltrate the endocardium, myocardium, and epicardium of the irradiated heart. They are the first responders releasing pro-inflammatory cytokines (e.g., monocyte chemotactic protein [MCP], TNF-α, and IL-8) to recruit other inflammatory cells [5, 43, 59] (Fig. 2). Recruited inflammatory cells may release further pro-inflammatory (e.g., IL-1, and IL-6) and pro-fibrotic cytokines including, e.g., TGF-β, connective tissue growth factor (CTGF), platelet-derived growth factor (PDGF), and fibroblast growth factor (FGF) [43, 58, 60–62]. Additionally, adhesion molecules, inflammatory and pro-fibrotic cytokines (e.g., ICAM-1, IL-6, and FGF) can also be produced by the microvascular endothelial cells suggesting their role in the maintenance of the pro-inflammatory state [35, 63–65]. Matrix metalloproteinases (MMPs), including MMP-1 and MMP-2, could immediately be activated by RT in endothelial cells, possibly via the increased oxidative/nitrosative/nitrative stress or inflammatory mechanisms [43, 58, 66, 67]. These proteases may degrade the endothelial basement membrane, allowing effective recruitment of neutrophils and macrophages to cellular injury sites in order to phagocyte tissue debris [43, 68] (Fig. 2). The recruited inflammatory cells and the damaged endothelial cells can produce a large amount of O_2_^•−^ and ^•^NO, the latter via iNOS, which results in further ONOO^−^ formation [58, 69]. The decreased bioavailability of ^•^NO could lead to endothelial damage, vascular dysfunction, vasoconstriction, and tissue hypoxia [35]. The mechanisms described above may lead to focal endothelial denudation and endothelial dysfunction, triggering initial arteriosclerotic lesions in larger coronary arteries [35].

#### Increased thrombogenicity and acute inflammation in RIHD

Initial endothelial damage in the microvasculature could also activate the coagulation cascade leading to fibrin deposition [43] (Fig. 2). Notably, it can be a result of (i) RT-induced endothelial damage itself [20], (ii) the thrombomodulin inhibition caused by TGF-β [70], and (iii) increased release of von Willebrand factor (vWF) from endothelial cells [35]. Several coagulation factors (e.g., thrombin) may induce the endothelial release of IL-8 and MCP, promoting the expression of adhesion molecules and chemotaxis of neutrophil granulocytes [71]. RT can also activate COX and LOX enzymes, which produce bioactive eicosanoids from arachidonic acid, including, e.g., prostaglandins, prostacyclin, thromboxanes, and leukotrienes in different cell types [72]. These bioactive molecules are well-known mediators of inflammation via vasodilation or vasoconstriction, vascular permeability, extravasation of leukocytes, and microthrombus formation [72]. Decreased bioavailability of ^•^NO can lead to vasoconstriction, aggravating thrombogenicity [35].

#### Endoplasmic reticulum stress and apoptosis in RIHD

Irradiation can induce endoplasmic reticulum stress (ERS) and cell death in the different cell types of cardiac tissue, including cardiomyocytes, endothelial cells, fibroblasts, and cells of the conducting system [35] (Fig. 2). RT-induced irreversible damage in the structure of cellular compartments and molecules, mitochondrial dysfunction, and ERS are the critical components in cell death pathways in RIHD [14]. During ERS, the ER is overwhelmed with incorrectly folded or unfolded proteins [73]. The protein overload induces disruption of protein homeostasis and activates the unfolded protein response (UPR). The UPR leads to apoptotic cell death via three major pathways [73]. These are (i) the protein kinase R-like endoplasmic reticulum kinase (PERK)-regulated, (ii) the activating transcription factor 6 (ATF6), and (iii) the inositol-requiring enzyme 1 (IRE1) pathways [74]. PERK inhibits protein translation via phosphorylation and subsequent inactivation of the eukaryotic translation initiator factor 2α (eIF2-α) to avoid further misfolded protein accumulation. The ATF6 and IRE1 pathways activate transcription of genes involved in ER-associated protein degradation, protein folding, and ER membrane expansion. IRE can also inhibit the anti-apoptotic activity of Bcl-2 and Bcl-XL [74]. After irradiation of cardiomyocytes, the stimulated ER releases calcium ions into the cytoplasm, leading to mitochondrial calcium overload, cytochrome-C release into the cytoplasm, and activation of the pro-apoptotic Bax [75, 76] (Fig. 2). The translocation of Bax from the cytoplasm to the mitochondrial outer membrane induces mitochondrial membrane permeability transition (MPT), leading to mitochondrial swelling, depolarization of the membrane, uncoupled electron transport, and oxidative phosphorylation [75] (Fig. 2).

#### Necrosis in RIHD

Several death initiators, signaling pathways, and effector molecules are common key mediators in both apoptosis and necrosis [77]. Triggered by elevated oxidative/nitrosative/nitrative stress and calcium ion toxicity after RT, the MPT is a causative event in cell death mechanisms, including both apoptosis and necrosis in cardiomyocytes [14]. Extrinsic stimuli via cell surface death receptors, such as TNF-α, Fas, and TNF-related apoptosis-inducing ligand (TRAIL) receptors, can also stimulate both types of cell death [77] (Fig. 2). Necrotic cells release factors like endogenous mitochondrial damage-associated molecular patterns (DAMPs), including high mobility group box 1 (HMGB1), ATP, and IL-1α produced by stressed cells to evoke an inflammatory response [78, 79]. These signals are sensed by the nucleotide-binding domain and leucine-rich-repeat-containing family pyrin 3 (NLRP3), a core protein of the inflammasome. NLRP3 activates and releases the pro-inflammatory cytokine IL-1β and IL-18. RT may activate NLRP3 inflammasome via multiple other mechanisms, including increased oxidative/nitrosative/nitrative stress, calcium ion influx, and potassium ion efflux [78, 79]. NLRP3 inflammasome was suggested to play a critical role in the development of RIHD via its complex relationship with cell death and inflammatory processes [78, 79].

#### Autophagy in RIHD

Autophagy, a predominantly cytoprotective catabolic process, has been linked to apoptosis and necrosis, providing either a pro-survival or pro-death function [80]. The homeostatic role of autophagy is particularly critical in terminally differentiated cells, including cardiomyocytes. Cytosolic components or cell organelles are packed into double membraned autophagic vesicles that fuse with lysosomes. It results in the degradation and recycling of cellular components, thereby promoting survival [77]. However, the over-activation of autophagy could be harmful under pathological conditions. RT can directly or indirectly damage DNA, which activates repairing signaling pathways. Many proteins participating in DNA damage repairing signaling pathways, such as p53, ATM, PARP1, FOXO3a, mTOR, and SIRT1, are involved in the regulation of autophagy (Fig. 2). Irradiation may also damage extranuclear targets such as plasma membrane, mitochondria, and ER, leading to increased ceramide, ROS/RNS, and calcium ion concentrations, which can activate many autophagic pathways [81, 82].

### Pathomechanisms in the chronic phase of RIHD

#### Mechanisms of the increased oxidative/nitrosative/nitrative stress in the chronic phase of RIHD

The early biochemical modifications, which occur during or shortly after the radiation exposure, were thought to be responsible for most of the effects of ionizing radiation in cells [48]. The initial oxidative/nitrative/nitrosative stress is caused by the radiolysis of water induced by ionizing radiation (see also Sect. “Mechanism of the increased oxidative/nitrosative/nitrative stress in the chronic phase of RHID). However, oxidative and nitrative changes might continue to present months or years after the initial radiation exposure, presumably due to continuously increased generation of ROS/RNS via different mechanisms including, e.g., mitochondrial damage, inflammatory and cell death processes (see also Sect.“The interplay of oxidative /nitrosative/nitrative stress with chronic inflammatory pathway in RIHD”.), overexpression of ROS-generating enzymes in cardiac tissue and insufficient antioxidant mechanisms [20, 48] (Fig. 3). Remarkably, these processes occur both in the irradiated cells and their progeny [48, 49]. It is also well-known that aging, cardiovascular risk factors (e.g., hypertension, diabetes mellitus, hypercholesterolemia, and chronic kidney disease) [83–85], and concomitant anthracycline therapy [86] are also associated with increased oxidative/nitrosative/nitrative stress and low-grade chronic inflammation (Fig. 3).

These factors might further aggravate the progression of RIHD.

#### The interplay of oxidative/nitrosative/nitrative stress with chronic inflammatory pathways in RIHD

In the chronic phase of inflammation, the elevated ROS/RNS levels may result in increased expression and activity of TGF-β [58]. TGF-β and other growth factors (e.g., CTGF and PDGF) promote myofibroblast differentiation, cardiomyocyte hypertrophy, the proliferation of endothelial cells and fibroblasts, leading to compensatory hypertrophy with increased collagen deposition and remodeling in the heart and vessel walls, and also stenosis in the vessel lumen [58]. With the exacerbated atherosclerosis and reduced capillary network, these processes may lead to myocardial hypoxia and chronic ischemia, potentially resulting in cell death and, ultimately, HF or IHD [87] (Fig. 3). Evidence suggests that chronic activation of the renin–angiotensin–aldosterone system (RAAS) and the sympathetic nervous system in HF also stimulates the inflammation and oxidative/nitrosative/nitrative stress, which factors further aggravate each other [88, 89]. Angiotensin II has been reported to activate cardiac NADPH oxidase and, subsequently, the overproduction of ROS/RNS. The increased oxidative/nitrosative/nitrative stress triggers the production of pro-inflammatory mediators, including, e.g., IL-1, IL-6, TNF-α, and TGF-β, contributing to cardiac remodeling and HF [90, 91] (Fig. 3).

#### Compensatory cardiac hypertrophy in RIHD

After RT-induced acute cell damage and death, a compensatory hypertrophy is initiated in the surviving cardiomyocytes to compensate for declined cardiac function due to the loss of cardiomyocytes. Chronic hypoxia, inflammatory pathways, and repetitive ischemia could play a role in the development of compensatory hypertrophy [31–34] (Figs. 1 and 3). Irradiation induces a significant increase in left ventricular wall thicknesses accompanied by reduced left ventricular inner diameters. In this early phase of HF, diastolic dysfunction develops with elevated left ventricular filling pressures and preserved ejection fraction (HFpEF) [33, 34, 92, 93]. It has also been reported that miR-212 [34], increased oxidative stress, TGF-β signaling, and exchange protein activated by cAMP (Epac) could play an essential role in the development of cardiac hypertrophy after RT [32, 33]. Epac was shown to increase intracellular Ca^2+^ flux, activate hypertrophic signals such as the Ca^2+^/calmodulin-dependent protein kinase II (CaMKII) and calcineurin, and induce fetal gene reprogramming independently of TGF-β-mediated fibrotic pathways [32].

#### Cardiac fibrosis in RIHD

Cardiac fibrosis is considered the main late cardiac side effect of RT, leading ultimately to HF with reduced ejection fraction (HFrEF) in the decompensated phase of HF (Figs. 1 and 3). It begins early after RT in parallel with the compensatory hypertrophy and might remain asymptomatic for years [39] (Figs. 2 and 3). Cardiac fibrosis is the result of abnormally increased extracellular deposition of collagen. The initiation stage of fibrogenesis is driven by the RT-induced primary vascular endothelial cell injury. The acute changes, occurring within a few hours after RT, are related to cell death and the resulting release of acute-phase inflammatory response molecules (e.g., PDGF, TGF-β, basic FGF, insulin-like growth factor [IGF], CTGF, IL-4, IL-13, IL-8, and MCP) [20, 43, 58]. The duration of the acute phase may be up to several days after the RT. Within 2–3 weeks after RT, fibrogenic effector cells, including fibroblasts, fibrocytes, tissue-specific pericytes, and myofibroblasts, are activated to differentiate into mature myofibroblasts in the second phase of fibrogenesis [58, 94]. The activated and terminally differentiated myofibroblasts secrete a high amount of type I, III, and IV collagens, as well as α-smooth muscle actin, into the extracellular matrix [43]. In this process, TGF-β is considered a key factor in promoting the differentiation and mesenchymal cells to myofibroblasts. TGF-β can activate the canonical SMAD2/3 and the non-canonical Rho/Rack pro-fibrotic pathways, inhibit the collagenases, and stimulate the production of CTGF [20]. In the third phase of fibrogenesis, myofibroblasts produce a large range of extracellular proteins, primarily in an autocrine manner, which may last several weeks or months after the second phase. Myofibroblasts are permanently activated in the irradiated tissues even after repair of the initial injury. This process is driven mainly by TGF-β and PDGF [95]. Another factor is the ROS/RNS-induced activation of the transcription factor NF-κB. Its activation results in increased adhesion molecule, cytokine, and chemokine production [43] (Fig. 3). NF-κB was shown to be chronically upregulated in irradiated human arterial vascular cells from 4 to 500 weeks after RT suggesting that it might play a critical role in the transition from acute to chronic inflammation and fibrosis [52]. The last phase of fibrogenesis is the manifest myocardial fibrosis developing years or decades after the RT. The progressive and diffuse interstitial fibrosis leads to decreased tissue elasticity and contractility as well as chronic hypoxia by separating and replacing the cardiomyocytes. [20, 35]. Cardiac fibrosis ultimately results in cell death mechanisms, organ dysfunction, and HF leading to a decompensated stage (Fig. 3).

#### Cellular senescence in RIHD

Cellular senescence was traditionally considered a process to inhibit uncontrolled replication in proliferative cells [96]. Nowadays, it is thought that post-mitotic cells also develop a senescent-like phenotype [96]. Generally, senescent cells become flattened, enlarged, and irreversibly lose the ability of proliferation [96]. Senescent cells produce increased levels of ROS/RNS, which represent increased oxidative/nitrosative/nitrative stress to neighboring cells. Typical senescent cells secrete a plethora of inflammatory mediators (e.g., cytokines and chemokines) and extracellular proteases, and the entity is named the senescence-associated secretory phenotype. This phenotype leads to chronic sterile inflammation and contributes to tissue remodeling [96, 97]. The role of senescent endothelial cells seems to be crucial in the development of RIHD (Fig. 3). RT-induced endothelial senescence may involve the activation of IGF1/phosphatidylinositol-3-kinase (PI3K)/Akt-mTOR pathway acting upstream of p53/p21, p38, NF-κB, and TGF-β type 1 receptor ALK5. The induction of ERS and repression of telomerase reverse transcriptase are also characteristics of senescent endothelial cells [96, 97]. Due to the decreased NO bioavailability, senescent endothelial cells are incapable of regulating vasodilation, resulting in accelerated atherosclerosis and hypertension (Fig. 3). They are pro-inflammatory, pro-thrombotic, and pro-atherogenic due to their increased production of various inflammatory cytokines, adhesion molecules, and plasminogen activator inhibitor-1 (PAI-1), and decreased levels of thrombomodulin (Fig. 3). They are also incapable of regeneration, leading to reduced density of cardiac capillaries and small coronary arterioles. The capillary rarefaction can lead to chronic cardiac hypoxia contributing to the RT-induced HF and IHD [96, 97].

## Diagnosis and follow-up of RIHD in patients

If needed, the early diagnosis and control of RIHD are essential since medical therapy or intervention may be of benefit. Control of RIHD should start with identifying individuals at risk for RIHD by registering heart and coronary artery dosimetry data. The existence of other cardiac risk factors such as history, age, and the use of chemotherapy, should be registered. In individuals at risk, clinical history and baseline measurement of cardiac function should be recorded. Regular monitoring of symptoms/signs and cardiac function should start after RT, while in non-risk patients, the patient's and physician's alertness is sufficient [13, 24]. There has been an interest in testing various biomarkers of myocardial injury or HF, including troponin I, troponin T, B-type natriuretic peptide, or inflammatory cytokines such as growth differentiation factor-15 and C-reactive protein. These may be used for the detection and follow-up of HF most often related to medical therapies, but only as a complementing tool of other diagnostic tools [98]. Cardiac imaging methods include echocardiography, nuclear imaging, cardio-CT, or MRI to serve better diagnosis [13]. Nevertheless, 2D speckle tracking echocardiography seems to be a sensitive and highly specific clinical approach for the detection of early subclinical heart abnormalities [98]. Cardiac SPECT perfusion scanning was reported to detect injury as early as a few months after RT [28]. The choice of the method may also depend on availability. For consistency reasons, the same expert is preferred to follow the case using the same diagnostic method. Different guidelines exist for the management of RIHD, including the follow-up of cancer patients after RT [12, 13, 21, 24, 99, 100]. During follow-up, careful exploration of symptoms (notably, radiogenic IHD presents relatively often silent), repeated ECG, stress and contrast echocardiography, 3D echocardiography, stress perfusion imaging, tissue Doppler imaging, and screening for coronary calcium deposits might be applied.

**Fig. 4:**
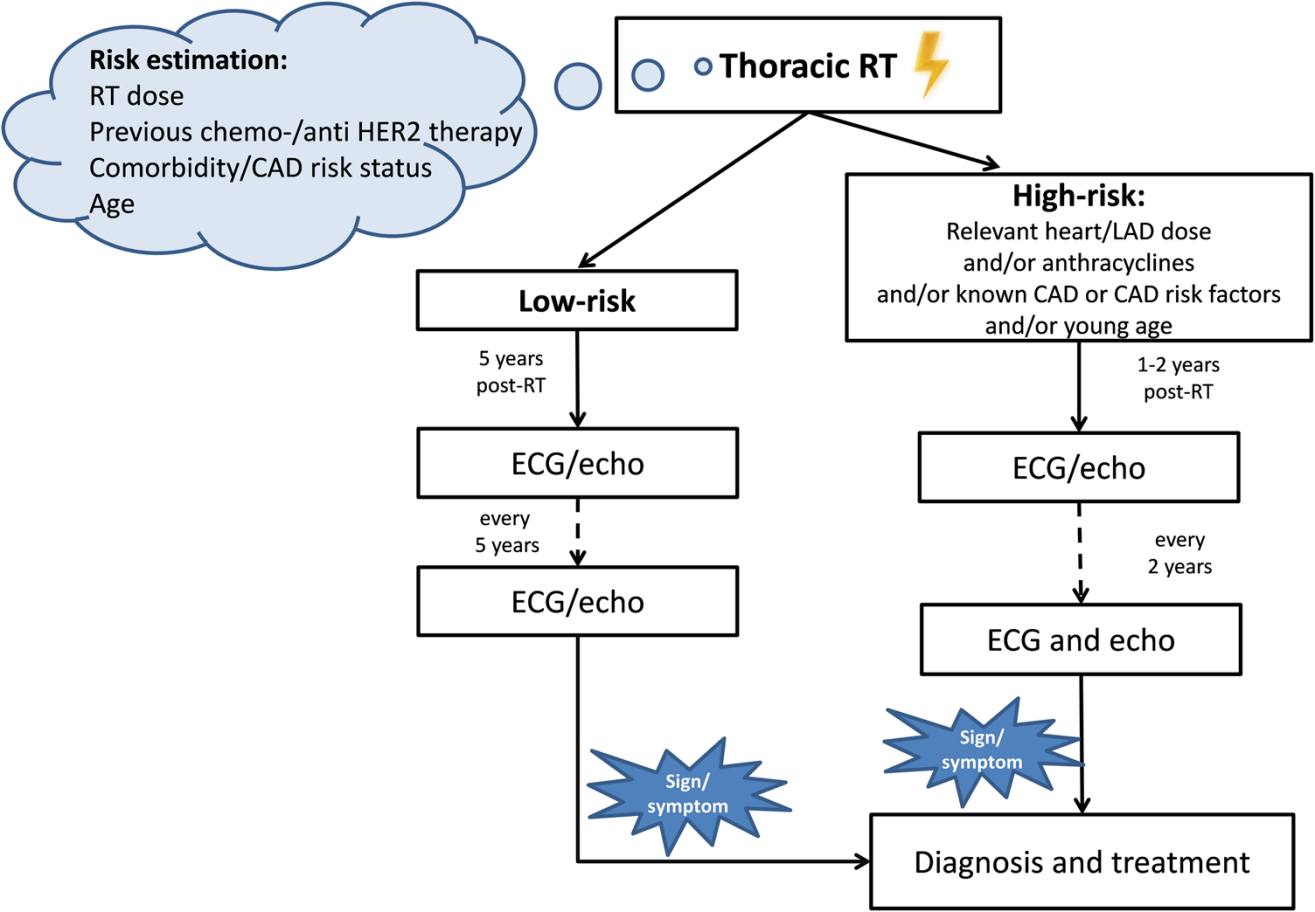
Algorithm of cardiovascular follow-up after thoracic RT. *CAD* coronary artery disease, *ECG* electrocardiography, echo: echocardiography, *HER2* human epidermal growth factor receptor, *LAD* left anterior descending artery, *RT* radiotherapy

Onco-cardiology follow-up in everyday practice is shown in Fig. 4. All guidelines recommend the specification of the risk-status, preferably before starting oncological treatments or before outlining individual follow-up strategies [12, 13, 24]. For that, heart dose, the use of toxic oncological treatments, comorbidities, and age should be considered. According to the risk level, follow-up should start immediately or many years after the RT. In high-risk patients (a mediastinal dose of > 30 Gy, or if anthracyclines were also given, etc.) monitoring should start 1–2 years post-irradiation and continue every 2 years thereafter; a great emphasis should be given to correcting risk factors. In low-risk cases, regular follow-up performed every 2–5 years started 5 years after the RT is considered sufficient. For the routine follow-up of asymptomatic patients, ECG and echocardiography are used. During follow-up, careful attention should be paid to medical history; if symptoms or suspicious signs develop, imaging and functional studies should be performed as previously described [12, 13, 24].

## Prevention and therapy of RIHD

Currently, there are two main strategies to lessen the burden caused by radiogenic heart sequelae. One is to prevent heart exposure as much as possible by applying new RT technologies and protocols [101]. Second, identifying those risk patients whose surveillance after RT is crucial, sometimes with comprehensive multimodality imaging-based screening protocols if necessary; the early diagnosis and non-specific management of radiation heart damage may effectively improve outcomes. A third possibility would be the application of pharmacons to protect the heart from radiation-induced damage. However, at present, no specific pharmaceutical agent is approved for the prevention or treatment of RIHD in the clinics. Nevertheless, several lines of evidence obtained in preclinical or clinical studies suggest that a number of pharmacological agents might be effective for the treatment of RIHD.

### Prevention of RIHD by cardiac dose-sparing techniques

It is mandatory to individually estimate the net benefit of RT before its start by balancing the gains against the greatness of cardiac dose and the patient's background cardiovascular risk in the absence of RT [102]. There are many approaches to protect the heart from radiation exposure in breast cancer patients [102]. Prone positioning during RT reduces heart doses in about 2/3 of the patients [103–105], while the deep-inspirational breath-holding (DIBH) technique is advantageous in an even higher proportion of patients [106, 107]. Both methods operate by separating the heart and the radiation fields. Intensity-modulated radiation therapy (IMRT) and proton irradiation are advanced techniques not yet widely applied while reducing the volume to be irradiated during partial breast irradiation (PBI), or the omittance of RT are options in low-risk of cancer recurrence cases. For the best risk–benefit ratio, the selection of individually tailored techniques and RT modality is needed [103, 104]. In non-breast cancer patients needing RT to the chest, developed techniques such as the IMRT/volumetric modulated arc radiotherapy (VMAT), cyberknife, or stereotactic radiosurgery are all based on image-guidance and particle RT. Sometimes breathing-control/gating ensure precise targeting and best protection of normal tissues [106]. Special consensus guidelines stressing the use of modern techniques, and selective RT have been elaborated for the modern RT of lymphoma patients [108]. RT guidelines with similar concepts have been published for lung cancer [109] and oesophageal cancer [110].

### Prevention and therapy of RIHD with pharmacological agents

Although many agents have been tested for the prevention of radiation damage, none of them yet gained registration with this indication. Most of our knowledge in this field is experimental only. The group of the so-called radioprotectors has anti-oxidant and/or anti-inflammatory properties being administered as molecular preventive strategies before radiation exposure [44]. The so-called mitigators are administered during or shortly after the irradiation with the aim of ameliorating the radiation injury of normal tissues [44]. A third approach is starting the protectant several weeks after the radiation exposure. The pharmacological treatment of RIHD initiated after the completion of the RT has a clear benefit with the advantage of not interfering with the efficacy of cancer therapy but, only a few preclinical studies tested this approach. Despite the lack of specific treatments for RIHD in clinical practice, some recommendations exist on using standard therapies in the radiation heart sequelae indication (e.g., HF or IHD) [12, 13]; furthermore, some promising novel approaches also exist. This section collects well-known drugs and promising novel agents mostly tested in rodent RIHD models. The description of the potential side effects of the well-known drugs is out of the scope of this review.

#### Anti-oxidants

Since the production of ROS/RNS is a crucial element in the development of acute and chronic RIHD, testing anti-oxidants seems logical.

*α-tocopherol (vitamin E)*

Administration of a single dose of tocotrienols 24 h before the cardiac irradiation preserved the Bax/Bcl2 ratio and prevented mitochondrial permeability transition and RT-induced alterations in the mitochondrial respiration in rats 2 weeks after RT [111] (Table 2). However, the single dose of tocotrienols could not improve the cardiac remodeling 28 weeks after RT [111]. In another study, the phosphodiesterase inhibitor pentoxifylline plus α-tocopherol given daily and started 1 week before the RT or 3 months after the RT reduced the collagen deposition in rats 6 months after RT [93] (Table 2). Daily administration of α-tocopherol plus pentoxifylline for 6 months started 3 days before the RT reduced collagen deposition and TGFβ1 levels in a rat model 6 months after the RT [112] (Table 2). However, the daily administration of α-tocopherol plus pentoxifylline for 3 months started 3 days before the RT could not reduce the cardiac remodeling 6 months after the RT [112] (Table 2). In another study, the daily application of pentoxifylline and α-tocopherol 3 months after the RT did not alter cardiac fibrosis and left ventricular expression of vWF, neuregulin-1, hypertrophic, and fibrotic signal mediators in rats 6 months after the RT [113]. However, the cardiac number of macrophages and mast cells was reduced [113] (Table 2).

**Table 2:** Potential protective agents against the development of RIHD based on preclinical evidence

Mode of action	Pharmacon(s)	Route of administration and dose	Timing of pharmacological treatment	FUP time after RT	Protective effects of pharmacons	Animal model of RIHD	Age/BW at start of experiment	RT dose (Gy)	Type of RT	Refs.
Antioxidants	Tocotrienols	Per os 230 mg/kg/	Start: 24 h before RT (single dose)	2 or 28 wk	↓ Bax/Bcl2 (both FUP times) ↓ mPTP opening (2 wk) No effect on remodeling (28 wk)	Male ratsSD rats	250–290 g	21 Gy	Local heart irradiation	[111]
Antioxidants	Pentoxifylline + α-tocopherol	Per os 100 mg/kg/d + per os 20 IU/kg/d	Start: 1 wk before RT or 3 mo after RT end: 6 mo	6 mo	In both timing: ↓ LV diastolic pressure, ↓ Col-I and Col-III deposition	Male SD rats	180–200 g	5 × 9 Gy daily	Local heart irradiation	[93]
Antioxidants	Pentoxifylline + α-tocopherol	ip. 15 mg/kg/d + ip. 5.5 mg/kg/d	Start: 3 days before RT end: 12 or 24 wk	24 wk	Only after 24 wk treatment: ↓ cardiac TGF-β1 (mRNA) ↓ Cardiac fibrosis	Male SD rats	12–14 wk	20 Gy	Local heart irradiation	[112]
Antioxidants	Pentoxifylline alone or with tocotrienols (containing α-tocopherol)	Per os 95–110 mg/kg/d ± per os 43 mg/kg/d (15–17 mg/kg/d)	Start: 3 mo after RT end: 6 mo	6 mo	↓Cardiac inflammatory infiltration: ↓ Mast cells and macrophages	Male SD rats	220–260 g	21 Gy	Local heart irradiation	[113]
Antioxidants	amifostine	ip. 200 mg/kg (single dose)	Start: 20–30 min before RT	24 hor 100 d	After 100 d: ↓ Myocardial degeneration (focal vacuolization)	MaleWistar rats	12–14 wk (240–260 g)	15 Gy	Local heart irradiation	[117]
Antioxidants	amifostine	ip. 200 mg/kg (single dose)	Start: 30 min before RT	6 mo	↓ Vascular injury ↓ vasculitis	Male Wistar rats	10–12 wk (200–250 g)	18 Gy	Local heart irradiation	[114]
Antioxidants	amifostine	ip. 160 mg/kg (single dose)	Start: 15–20 min before RT	6 mo	in the 22.5 Gy group: maintained cardiac output ↓ Ventricular fibrosis	Female SD rats	12 wk old	15, 20 or 22.5 Gy	Local heart irradiation	[118]
Steroidal anti-inflammatory drugs	Dexamethasone	iv. 0.5 mg/d	2 h before RT and identical dose every 24 h for 3 d	24 h or 100 d	↓ Cardiac OH-Pro content ↓ Cardiac fibrosis	Male New Zealand rabbits	2.2–2.6 kg	20 Gy	Local heart irradiation	[121]
Steroidal anti-inflammatory drugs	Methylprednisolone	iv. 30 mg/kg	2 h before RT and then twice daily for 3 d	100 d	↑ Survival ↓ Cardiac OH-Pro content ↓ Cardiac fibrosis ↓ Pericarditis and pericardial effusion	MaleNew Zealand rabbits	1.8–2.4 kg	20 Gy	Local heart irradiation	[122]
Non-steroidal anti-inflammatory drugs	Ibuprofen	iv. 12.5 mg/kg	2 h before RT and then twice daily for 2 d	100 d	↑ Survival ↓ Cardiac OH-Pro content ↓ Cardiac fibrosis ↓ Pericarditis and pericardial effusion	Male New Zealand rabbits	1.8–2.4 kg	20 Gy	Local heart irradiation	[122]
Cardioprotective drugs	Trimetazidine	Per os 10 or 20 mg/kg/d	Start: 1 wk before or 1 wk after RT end: 8 wk	8 wk	↓ Fibrosis via the CTGF/TGF-β1/Smad pathway	Male C57BL6 mice	8 wk (18–22 g)	20 Gy	Thoracic irradiation	[127]
Cardioprotective drugs	GHRH and its agonistic analouges (JI-34 and MR-356)	1,10, 50, 100 or 500 nM	Start: after RT	48 h	↑ Viablity ↓ ROS level ↓ Activity of RISK/SAFE pathway (only JI-34)	neonatal rat cardiomyocytes	–	10 Gy	–	[134]
Statins	Lovastatin	Per os 10 mg/kg	Pre-treatment only 48 h and 24 h before RT	4–24 h or 3 wk	↓ Cardiac expression of NF-KB ↓ Cell adhesion molecules ↓ Pro-inflammatory markers ↓ Pro-fibrotic markersin a time-dependent manner	Female Blb/c mice	3–4 mo (25 g)	6 Gy or 2 × 2.5 Gy	TBI	[141]
Statins	Atorvastatin	Per os 0.25 mg/d	Start: 1 d before RT end: 6 wk	6 wk	↓ LV conexin-43 ↓ LV PKCε ↓ LV miR-21	MaleWistar rats	3 mo	25 Gy	Local heart irradiation	[138]
Statins	Atorvastatin	10 or 20 mg/kg/d	Start: 12 wk before RT or from first d after RT end: 12 wk	12 wk	↑ EF and FS ↓ cardiac fibrosis via the TGF-b1/Smad3, RhoA/ROCK, and PI3K/AKT pathways	Male SD rats	11–14 wk	7 × 3 Gy	Local heart irradiation	[142]
RAAS inhibitors	Captopril	Per os 30–60 mg/kg/d	Start: first day after RT end: 3 mo	3 mo	↓ LVEDP ↓ Pericardial fibrosis ↓ Cardiac fibrosis	Male Wistar rats	–	20 Gy	Local heart irradiation	[144]
RAAS inhibitors	captopril or enalapril or fosinopril	Per os 145–207 mg/m2/d per os 19–28 mg/m2/d per os 19–28 mg/m2/d	Start: 1 wk after RT end: 7 mo	7 mo	↓ Pulmonary collagen synthesis	Female WAG/RijCmcr rats	9–10 wk	13 Gy	Thoracic irradiation	[145]
Novel pharmaceutical agents	rhNRG-1β	iv. 15 μg/kg	3 d before RT + 7 d after RT	20 wk	↑ FS ↑ Mitochondrial function ↓ Cardiac fibrosis via the ErbB2-ERK SIRT1 pathway	Male Sprague–Dawley rats	200–250 g	21 Gy	Local heart irradiation	[151]
Novel pharmaceutical agents	Ac-SDKP	sc. 3.2 mg/kg/d	Start: within 24 h after RT end: 18 wk	18 wk	↓ Inflammation ↓ Macrophage activation ↓ Fibrosis	Male and female SD rats	10–12 wk	30 Gy	Local chest irradiation	[156]
Novel pharmaceutical agents	PACAP38	ip. 10 µg/100 µl	Start: 2 h before RT + additional doses: 24 h and 48 h after RT	21 days	↓ Apoptosis ↓ Cardiac fibrosis	Male C57/BL6 mice	6–8 wk (20–25 g)	14 Gy	Thoracic irradiation	[159]
Novel pharmaceutical agents	IPW-5371 (TGF-β1 receptor inhibitor)	Per os 10 or 30 mg/kg/d	Start: 24 h after RT end: 6 or 20 wk	6 mo	↑ Survival ↑ Cardiac function ↓ Cardiac fibrosis	Male and female C57L/J mice	6–8 wk	11.5 Gy	5 Gy TBI + 6.5 Gy thoracic irradiation	[162]

*Amifostine (Ethyol)*

The inactive prodrug amifostine (WR-2721) is a phosphorylated thiol, which can be converted to its active form (WR-1065) by alkaline phosphatase-catalyzed dephosphorylation in the vascular endothelial cells [114]. It has been demonstrated that amifostine protects normal tissues from both acute and chronic damage without interfering with the effects of RT on the tumor [115, 116]. Several preclinical studies have shown that a single dose of amifostine given 15–30 min before irradiation could be protective against RT-induced cardiac fibrosis, myocardial dysfunction, and vascular damage 100 days or 6 months after RT [114, 117, 118] (Table 2).

*Natural products*

Several natural products, including hesperidin, curcumin, melatonin, caffeic acid phenylethyl ester, black grape juice, and the ginger component zingerone, have been proposed as radioprotective agents due to their antioxidant and/or anti-inflammatory properties against RIHD as reviewed recently [119].

#### Anti-inflammatory drugs

Inflammation plays a major role in the development of RIHD, so it is not surprising that attenuation of the inflammatory response may beneficially affect the cardiac consequences of RT.

*Colchicine*

Colchicine is known to inhibit microtubule polymerization. Therefore, it can inhibit mitosis, neutrophil motility, and decrease platelet aggregation. The anti-inflammatory and platelet aggregation inhibiting properties of colchicine are suggested to be protective against RIHD [120]. However, there is no experimental or clinical evidence available in the literature for its use in RIHD.

*Steroidal anti-inflammatory drugs*

Reeves et al. reported that the steroidal anti-inflammatory drugs, dexamethasone, and methylprednisolone given 2 h prior to heart irradiation and every 24 h for 3 consecutive days reduced the cardiac fibrosis and hydroxyproline content in male rabbits 100 days after RT [121, 122] (Table 2).

*Non-steroidal anti-inflammatory drugs (NSAIDs)*

Administration of the non-selective NSAID ibuprofen administered 2 h prior to heart irradiation and for 2 days thereafter reduced fibrosis, pericarditis, pericardial effusions, and improved survival in male rabbits 100 days after RT [122] (Table 2). It has been reported that the use of various several non-selective COX inhibitors (e.g., ibuprofen, diclofenac, and naproxen) and selective COX-2 inhibitors (e.g., celecoxib) increased the risk of AMI [123]. Therefore, several NSAIDs are contraindicated among patients with CVDs. However, there is limited data available about the effects of NSAIDs on the risk of AMI in patients treated with thoracic RT. Uehara et al. investigated the effects of NSAIDs, including diclofenac, etodolac, indomethacin, ketoprofen, meloxicam, and rofecoxib, on RT-induced expression of ICAM-1, VCAM-1, E-selectin, and COX-2 in human umbilical vein endothelial cells (HUVECs) [124]. They found that indomethacin, diclofenac, and meloxicam given 1 h before RT highly upregulated the RT-induced expression of ICAM-1 and COX-2 in HUVECs, suggesting the potentiating effects of these NSAIDs on RT and the increased risk for AMI after thoracic RT [124]. The COX-2 inhibitor celecoxib has been reported to act synergistically with RT in cancer cells since it attenuates tumor growth and expression of cell proliferation markers and induces apoptosis in tumor cells [125]. The increased expression of adhesion molecules and apoptotic effects of celecoxib or several NSAIDs might be responsible for the increased risk for AMI after RT. However, further preclinical and clinical studies are needed to evaluate the effects of NSAIDs on the development of RIHD.

#### Cardioprotective drugs

Cardioprotective drugs are used to lower the severity of consequences due to CVD risk factors under various stress conditions. The application of such drugs in the case of chest RT may be useful.

*Trimetazidine*

Trimetazidine is an antianginal drug that inhibits the beta-oxidation of fatty acids by blocking mitochondrial long-chain 3-ketoacyl coenzyme A thiolase [126]. Trimetazidine also has anti-oxidant, anti-apoptotic, and inflammatory effects and may improve endothelial function [127]. Daily trimetazidine treatment started 1 week before or after chest irradiation reduced cardiac fibrosis via the CTGF/TGF-β1/Smad2/3 axis 8 weeks after the RT [127] (Table 2).

*Growth hormone-releasing hormone (GHRH) and its agonists*

The growth hormone-releasing hormone (GHRH) and its agonistic analogs are involved in the metabolism of reactive oxygen and nitrogen species and the proliferation and survival of a series of normal cells, including cardiomyocytes [128–130]. The administration of GHRH or GHRH analogs improved contractile recovery, ventricular remodeling during reperfusion, and reduced infarct size [131–133]. Similarly, two GHRH agonists, JI-34, and MR-356 showed increased viability and reduced ROS levels 48 h after irradiation in neonatal rat cardiomyocytes [134] (Table 2). However, only JI-34 could reduce the activity of the hypertrophic RISK/SAFE pathway [134] (Table 2).

#### Anti-atherosclerotic drugs, standard IHD and HF regimens

Recent position papers of the European Society of Cardiology and the German Cardiac Society summarize all the possible toxic effects of anti-cancer therapies on cardiovascular health comprehensively and stress the need for cardio-oncology services [12, 13]. Endothelial dysfunction and atherosclerosis play an important role in the development of RIHD. Age and cardiovascular risk factors can further accelerate the atherosclerotic process in long-term cancer survivors. Hence, the attenuation of endothelial dysfunction and atherosclerosis might lower the cardiovascular consequences of RT. Cancer patients presenting with clinical HF or IHD during or following cancer treatment need access to medical therapy with standard HF regimens or sometimes transcatheter or open surgical interventions [13]. Combined management with the control of age-related comorbidities and cardiovascular risk factors (such as hypertension, hypercholesterolemia, and diabetes) is crucial [135].

*Statins*

The widely used statins are blood cholesterol-lowering drugs that inhibit the key enzyme, 3-hydroxy-3-methylglutaryl coenzyme A (HMG-CoA) reductase, playing an important role in the endogenous cholesterol synthesis [84]. Therefore, statins are used in the treatment of hypercholesterolemia and atherosclerosis [84]. Recent studies showed that statins could also reduce cardiac oxidative/nitrosative/nitrative stress, acute inflammatory reactions, and fibrosis via different mechanisms [136–138]. Notably, in RIHD, the inhibition of RhoA GTPase, which plays a crucial role in endothelial cell migration, might be of benefit [139]. Statins can also decrease the cardiac endothelial cell permeability via activating ERK5 and increase the release of the vasodilator NO [140]. Ostrau et al. showed that single doses of lovastatin given 48 and 24 h before RT reduced the expression of NF-κB, cell adhesion molecules, pro-inflammatory and pro-fibrotic markers in a time-dependent manner from 24 h to 3 weeks after RT in rats [141] (Table 2). In a rat model of RIHD, daily administration of atorvastatin started 1 day before RT decreased the expression of the profibrotic miR-21, connexin-43 and PKCε in the left ventricle 6 weeks after the RT [138] (Table 2). Zhang et al. reported that daily atorvastatin treatment started 3 months before or 1 day after the RT improved cardiac function and reduced fibrosis via the repression of TGF-β1, Smad3/p-Smad3, Rho/ROCK, p-Akt, and fibronectin in rats [142] (Table 2).

#### RAAS inhibitors

RAAS plays a pivotal role in the development of CVDs, including hypertension, atherosclerosis, AMI, cardiac hypertrophy, and HF, via different systemic and tissue-specific effects [143]. The common point in the pathophysiology of the various CVDs is the microvascular injury that leads to subsequent myocardial ischemia and late fibrous remodeling [143]. Clearly, the pathophysiology of RIHD is similar to that of other etiologies induced by angiotensin II and aldosterone. Cardiac concentrations of angiotensin II and aldosterone were dose-dependently increased 3 months after heart irradiation in rats [143]. Importantly, the daily administration of the angiotensin-converting enzyme (ACE) inhibitor captopril started on the first day after the RT ameliorated the perivascular and cardiac fibrosis as well as the diastolic dysfunction 3 months after the RT in rats [144] (Table 2). Another study demonstrated that daily treatments of three structurally-different ACE inhibitors, captopril, enalapril, and fosinopril, given 1 week after thoracic RT reduced pulmonary collagen synthesis 7 months post-radiotherapy in rats [145] (Table 2). Although these data are interesting, prospective studies evaluating the efficacy of ACE inhibitors in patients undergoing thoracic RT are lacking in the literature. The PRADA clinical trial enrolling 130 women with early breast cancer demonstrated that adjuvant, anthracycline-containing regimens with or without trastuzumab and RT were associated with a modest reduction of the left ventricular ejection fraction (LVEF). This decline in LVEF was significantly alleviated by the concomitant administration of the angiotensin receptor blocker (ARB) candesartan [146]. In contrast, a retrospective clinical study enrolling 76 patients undergoing coronary artery stenting after thoracic RT (> 30 Gy) found that ACE inhibitors or ARBs and higher MHD were related to lower overall survival since cancer diagnosis [26]. In order to clarify whether RAAS inhibitors may be protective against RIHD, clinical trials enrolling a large number of patients are required for widening the indication of this otherwise routinely used medication of heart failure.

#### Novel pharmaceutical agents

*Recombinant human neuregulin-1β (rhNRG-1β)*

Neuregulin-1β (NRG-1 β) binds to the tyrosine kinase receptors of the ErbB family, i.e., ErbB2, ErbB3, and ErbB4 in the heart [147]. NRG-1β is critical for cardiac development and repair, and recombinant forms are currently being assessed in clinical trials as possible therapeutic agents for systolic HF even [NCT03388593, NCT04468529]. Indeed, numerous preclinical studies confirmed the anti-inflammatory, anti-remodeling, and anti-fibrotic effects of NRG-1 in the heart [148–150]. RhNRG-1β given 3 days before and 7 days after heart irradiation decreased RT-induced myocardial fibrosis and cardiomyocyte hypertrophy while preserving cardiac function via the ErbB2-ERK-SIRT1 signaling pathway 20 weeks after the RT in rats [151] (Table 2). In preclinical studies, NRG-1 was also protective against doxorubicin-induced or trastuzumab-induced cardiotoxicity, a common unwanted side-effect related to drugs used in breast cancer patients [152, 153]. Although some studies have proposed that NRG1 may promote tumor growth, NRG1 could also act as a tumor suppressor in breast cancer [154, 155]. Further studies are needed to clarify the effects of rhNRG-1β on the tumor growth-stimulating potential of rhNRG-1β.

*N-acetyl-Ser-Asp-Lys-Pro (Ac-SDKP)*

Ac-SDKP is a ubiquitous endogenous peptide originating from thymosin-β4, which is essential for epicardial progenitor mobilization and neovascularization [156]. Ac-SDKP showed anti-fibrotic and macrophage inhibitory effects via inhibiting the expression and activity of a carbohydrate-binding surface protein, Mac-2 (galectin-3), in preclinical models [157, 158]. Ac-SDKP administered daily for 18 weeks after thoracic RT inhibited inflammation, fibrosis, and reduced macrophage activation in rats [156] (Table 2).

*Pituitary adenylate cyclase-activating polypeptide 38 (PACAP38)*

The endogenous peptide PACAP38 is a member of the secretin/vasoactive intestinal peptide (VIP) family. It has anti-inflammatory, anti-apoptotic, and antioxidant effects in cardiomyocytes [159, 160]. PACAP38 was radioprotective in H9c2 cardiomyoblasts via anti-apoptotic effects and the overexpression of the nuclear factor erythroid 2-related factor 2 (NRF2), which is a key factor in the expression of antioxidant enzymes [159]. PACAP38 given 2 h before irradiation, and at 24 h and 48 h after irradiation reduced apoptosis and cardiac fibrosis 21 days after the RT in male C57BL6 mice [159] (Table 2).

*Potential anti-inflammatory and anti-fibrotic biological therapies*

A common mechanism in the pathophysiology of HF and IHD of non-radiation etiology is chronic inflammation and fibrosis. So far, no anti-inflammatory or anti-fibrotic biological therapy has been tested in RIHD in clinical trials. However, some anti-inflammatory agents, including, e.g., the IL-1 receptor antagonist anakinra (clinical trials NCT03797001, NCT01175018, and NCT02547766) or the IL-6 receptor antagonist tocilizumab (clinical trial NCT01491074), have been already tested against post-infarction remodeling and HF in phase 2 clinical trials. Interestingly, the human anti-CTGF antibody (FG-3019) started at different time points before or after thoracic irradiation ameliorated the pulmonary remodeling in a mice model [161]. However, anti-CTGF antibodies have not been tested in the clinical phase in RIHD yet. The TGF-β receptor-1 antagonist IPW-5371 administered orally 24 h after the radiation exposure and daily after that for 6 or 20 weeks was reported to mitigate the injury of RT (5 Gy total body irradiation plus 6.5 Gy thoracic irradiation) in C57BLJ mice [162]. IPW-5371 treatment for both 6 and 20 weeks improved survival. The 20-week therapeutic regimen preserved arterial oxygen saturation with significant decreases in breathing frequency. It reserved the cardiac contractile function and ameliorated cardiac and pulmonary fibrosis [162]. IPW-5371 treatment decreased p-Smad3 tissue levels, confirming the effect of IPW-5371 on TGF-β signaling [162] (Table 2). IPW-5371 is not yet in the clinical phase, but another TGF-β1 receptor inhibitor, the orally bioavailable galunisertib (LY2157299), has already been tested in phase II clinical trials in metastatic breast cancer patients in combination with RT (NCT02538471), hepatocellular carcinoma (NCT02178358), and pancreatic cancer (NCT01373164).

#### Cardiac-specific drug delivery

Radioprotective agents may interfere with the anti-cancer effects of RT. Therefore, the development of cardiac-specific drug delivery techniques (e.g., liposomes, polymeric micelles, biodegradable nanoparticles, dendrimers, exosomes, plasmids, or other vectors) [163]. would be of interest in order to selectively protect the heart from ionizing radiation and thus preventing RIHD. Several drugs, including VEGF delivered by plasmids or adenoviruses to the heart, have already been tested in HF and AMI in clinical trials (NCT03409627, NCT01002430, NCT04125732, respectively). Unfortunately, up to now, no major breakthrough has been achieved in the field of targeted drug delivery for the prevention of RIHD.

### Gene expression regulators as potential new therapeutic targets: non-coding RNAs

The gene expression is regulated at different levels, including the post-transcriptional regulation by non-coding RNAs (ncRNAs). The ncRNAs are classified as *i*) longer ncRNAs such as long non-coding RNAs (lncRNAs) and circular RNAs (circRNAs), *ii*) and short ncRNAs (19–22 nucleotides) such as microRNAs (miRs) and small interfering RNAs (siRNAs) [164]. At present, literature data are available on the role of miRs only in the development of RIHD. miRs are thought to be tissue-specific fine-tuners of gene expression in biological processes, including those related to oxidative/nitrative stress, cell death, cell proliferation and development, and inflammation [165]. Hence, dysregulation of miRs in pathological processes, including RIHD, may alter whole gene networks rather than single genes. The major difference between miRs and siRNAs is that siRNAs are highly specific for one mRNA target, whereas miRs have multiple targets. Consequently, miR replacement or blockade with anti-sense inhibition therapy may offer a new approach to treat diseases by modulating complex gene pathways [166]. However, at present, the use of miRs as therapeutic targets or biomarkers has many limitations. A specific miR can target several mRNAs, and different miRs can target a particular mRNA in many tissue types. Therefore, potentially harmful effects of miR-based drugs (antagomiRs or agomiRs) could originate not only from off-target side effects (e.g., unwanted gene expression changes) but also from on-target side effects in non-targeted and non-diseased tissues [167]. In pre-clinical studies, cellular senescence-associated miR-34 [168], and pro-fibrotic miR-21 [169] were repressed, and the anti-fibrotic miR-15 [169] was overexpressed in the heart. These miRs seems to be promising in the future treatment of RIHD [138, 169]. Since the miR expression profile can be tissue-specific, the circulating miR profile may reflect the severity of radiation damage and correlate with the risk for the development of late organ-specific complications after RT. A clinical study enrolling breast cancer patients demonstrated the association between the circulating levels of the atherosclerosis-associated miR-146a, miR-155, miR-221, and miR-222 and later development of RT-induced cardiovascular complications [170]. Another clinical study found in non-small cell lung cancer NSCLC patients that the decrease of circulating miR-29a-3p and miR-150-5p levels was correlated with the RT dose delivered to the chest [171].

## Conclusions and perspectives

Much has been learned about the pathomechanism and clinical significance of RIHD in the past decades. All forms of radiation heart damage are dose-dependent and volume-dependent progressive alterations that should be prevented or, if manifest, should be early diagnosed and effectively treated to prevent definitive deterioration or fatal outcome. The key-event is cellular damage that results in cell loss and initiates inflammation and fibrosis, which may affect any of the structures of the heart. Although experimental in vitro and in vivo models have been used to study the pathophysiology of RIHD, the exact molecular mechanisms in the development of acute and chronic phases and the crosstalk between them are still not entirely clear. Future in vivo models that would better reflect clinical situations such as the presence of cardiovascular comorbidities or cancer, the modulatory effects of multimodality cancer therapy, and sex-based differences are much-needed. At present, there are no specific drugs approved for the prevention or treatment of RIHD. To lessen the burden that RT may cause to patients needing chest irradiation, the consideration and control of radiation dose to normal tissues and the use of individually selected RT techniques is essential. Obviously, prospective clinical trials are needed for the identification of novel predictors of radiation heart damage, new models, and new guidelines for optimal heart sparing. Future developments in cardiac-specific drugs or selective administration techniques, as well as personalized therapeutic approaches, are also essential for effectively reduce cardiovascular adverse effects following thoracic RT. Tailored monitoring of radiation heart sequelae based on individual risk-status within the frame of multidisciplinary onco-cardiology teamwork would enhance early intervention if needed.
